# Implementation of a digital nurse to improve the use of digital health applications (DiGA) for older people with depressive disorders (DiGA4Aged): a randomized proof of concept study

**DOI:** 10.1186/s13063-025-08809-7

**Published:** 2025-04-10

**Authors:** Anna Mai, Magdalena Pape, Theresa Sophie Busse, Katharina Kunde, Jennifer Bosompem, Chantal Giehl, Ina Carola Otte, Stephan Herpertz, Georg Juckel, Ida Haussleiter, Rainer Wirth, Horst Christian Vollmar, Nina Timmesfeld, Jan Dieris-Hirche

**Affiliations:** 1 Department of Medical Informatics, Biometry and Epidemiology, Ruhr University Bochum, Bochum, Germany; 2 Department of Psychosomatic Medicine and Psychotherapy, LWL-University Hospital, Ruhr University Bochum, Bochum, Germany; 3 Department of Clinical Psychology and Psychotherapy, University of Bamberg, Bamberg, Germany; 4 Institute of General Practice and Family Medicine (AM RUB), Ruhr University Bochum, Bochum, Germany; 5 Department of Health Services Research, Institute for Diversity Medicine, Ruhr University Bochum, Bochum, Germany; 6 Department for Geriatric Medicine, Marien Hospital Herne, University Hospital Ruhr University Bochum, Herne, Germany; 7 Department of Psychiatry, LWL-University Hospital, Ruhr University Bochum, Bochum, Germany; 8 Juniorprofessorship for Digital Health, Faculty of Health, Witten/Herdecke University, Witten, Germany

**Keywords:** Older adults, Depression, Digital health application, Digital health, Digital nurse, Assistance, Support, Digital therapy, mHealth, Health literacy

## Abstract

**Background:**

In the face of extensive waiting times for outpatient psychotherapy, prescriptible digital health applications (DiGA) are a useful and effective addition to the range of available therapy options for patients with mild to moderate depression. However, older adults face a particular challenge in implementing DiGA since higher age is a decisive predictor of lower digital health literacy. The necessity of an independent use of the prescribed DiGA is therefore associated with challenges for older patients and providers. In practice, it is crucial not to leave patients, especially older adults, alone after prescribing, but to maintain close contact to overcome technical and motivational barriers and to ensure that the novel application is used. However, this is difficult for physicians and psychotherapists due to the critical healthcare system situation in Germany described above. Another support system is needed. Hence, the main hypothesis of this study is that the additional implementation of digital nurses leads to a higher percentage of older patients with depressive symptoms starting DiGA use compared to a prescription and information alone.

**Methods:**

Two DiGA for mild to moderate depression in older patients were available and permanently approved at the time of the funding application. Using the most suitable one of them, as shown in a pilot study, the feasibility of implementation will be examined within a randomized proof of concept study. In our study, a digital nurse is trained to support patients with depression in using a DiGA. The main outcome is DiGA use (first session started: yes/no) after 8 weeks. Major secondary outcomes are patient-relevant outcomes, feasibility of recruitment and intervention, and factors moderating the effect or predicting DiGA use in the target group. Best practice guidelines will be elaborated on how to support and improve DiGA prescription and successful use in this population.

**Discussion:**

In Germany, the approved DiGA are currently little used, especially by people with a low digital affinity. This proof of concept study will use the example of older people with depressive disorders to show whether it is possible to increase the usage rate of a DiGA with the support of a digital nurse so that a DiGA can become a serious therapy option.

**Trial registration:**

DRKS: DRKS00033535. Registered on February 2, 2024.

## Administrative information

Note: the numbers in curly brackets in this protocol refer to SPIRIT checklist item numbers. The order of the items has been modified to group similar items (see http://www.equator-network.org/reporting-guidelines/spirit-2013-statement-defining-standard-protocol-items-for-clinical-trials/).

**Table:** 

Title {1}	Implementation of a digital nurse to improve the use of digital health applications for older people with depressive disorders (DiGA4Aged): a randomized proof of concept study.
Trial registration {2a and 2b}.	German Register for Clinical Trials (Deutsches Register für Klinische Studien, DRKS): DRKS00033535; registered on 02-02−2024; https://drks.de/search/de/trial/DRKS00033535.
Protocol version {3}	Version 4.0 of 15-07−2024.
Funding {4}	The study is funded by the FoRUM Innovation Fund (Grant Number: IF-025-22), Medical Faculty of Ruhr University Bochum; financial support.
Author details {5a}	AM, NT: Department of Medical Informatics, Biometry and Epidemiology, Ruhr University Bochum, Germany; MP, SH, JHD: Department of Psychosomatic Medicine and Psychotherapy, LWL-University Hospital, Ruhr University Bochum, Germany; MP: Department of Clinical Psychology and Psychotherapy, University of Bamberg, Germany; KK, TSB, JB, HCV: Institute of General Practice and Family Medicine (AM RUB), Ruhr University Bochum, Germany; IO: Department of Health Services Research, Institute for Diversity Medicine, Ruhr University Bochum, Germany; CG, RW: Department for Geriatric Medicine, Marien Hospital Herne, University Hospital Ruhr University Bochum, Herne, Germany; IH, GJ: Department of Psychiatry, LWL-University Hospital, Ruhr University Bochum, Germany; TSB: Juniorprofessorship for Digital Health, Faculty of Health, Witten/Herdecke University, Germany.
Name and contact information for the trial sponsor {5b}	Investigator initiated clinical trial; J. Dieris-Hirche (Principal Investigator); jan.dieris-hirche@ruhr-uni-bochum.de.
Role of sponsor {5c}	This is an investigator initiated clinical trial. Therefore, the funders played no role in the design of the study and collection, analysis, and interpretation of data and in writing the manuscript.

## Introduction

### Background and rationale {6a}

In the face of extensive waiting times for outpatient psychotherapy, digital health applications are a useful and effective addition to the range of available therapy options for patients with depression and other psychiatric disorders, which should be considered in treatment planning [1, 2]. Smartphone apps have been shown to reduce depressive symptoms in patients [3] and can therefore be used to support treatment or bridge waiting times [4]. Nuij et al. [5] examined smartphone apps in inpatient depression treatment and found that apps in addition to behavioral therapy-based psychotherapy improved the severity of depressive symptoms. This effect is also seen after discharge from inpatient treatment, underscoring the usefulness of digital applications in outpatient care as well.

In addition to general health apps, so-called DiGA (digital health applications) exist in Germany, which can be prescribed by physicians and psychotherapists. The prescription of a DiGA is possible at the expense of the statutory health insurance and in some cases also the private health insurance since the Digital Health Care Act (Digitale-Versorgung-Gesetz, DVG) came into force on 19th December 2019 [6]. According to a report of the Central Association of the Statutory Health Insurance (GKV-Spitzenverband), around 203,000 DiGA were prescribed and approved by the statutory health insurance funds in the period from the 1st of September 2020 to the 30th September 2022 (Central Association of the Statutory Health Insurance [7]).

DiGA are low-risk medical devices (class I or IIa). They have been tested for their safety, data protection, medical quality, performance, interoperability requirements, and positive care effects through scientific studies as part of a procedure conducted by the Federal Institute for Drugs and Medical Devices (BfArM) [8]. This is done in a so-called fast-track procedure, in which a decision is to be made after only 3 months. If the vote is positive, the DiGA are included permanently or temporarily (for 1 year) in the DiGA directory of the BfArM. In June 2024, 64 DiGA were listed in the DiGA database (https://diga.bfarm.de/de/verzeichnisExternalRef>), of which 35 are permanently included. Most DiGA are from the “mental illness” category (currently 27), thereof 7 target depressive disorders. At the time of study planning, the BfArM DiGA list included two permanently approved DiGA for the treatment of people with depressive disorders. One was approved for use in mild as well as moderate depressive disorders, the other had an approval for mild, moderate, and severe depressive disorders. Both DiGA are based on cognitive behavioral therapy (CBT) approaches, psychoeducation, self-monitoring, and emotion regulation exercises, which individuals go through independently via a web application or app. The programs are designed to be interactive and adapt to the user's responses and interactions (e.g., interactive dialog). Motivational emails are sent for support or basal email contacts with psychological counselors from the manufacturers are offered. Recommended durations of use are comparable and are approximately 90 days and 12 weeks, respectively. Weekly usage durations of 1–2 h are recommended. Our working group is currently testing in a pilot study which of the two DiGA is more suitable for people aged 60 and older in a qualitative pre-study (DRKS registration no. DRKS00033640, [9]). This DiGA will then be used in this RCT.

A survey of general practitioners published in 2020 revealed that many physicians had little experience using health apps in general at that time, and 63% of respondents also had a low or very low willingness to prescribe DiGA. It should be noted that 51% of respondents indicated that their knowledge of the DVG was somewhat poor, 12% rated it as poor, and 12% were unaware of the DVG. A study on experiences and observations of physicians regarding the use of DiGA in 2022 showed that the prescribed applications were rated as useful [10]. Positive care effects were observed, these concern the improvement of compliance, mobility, and education as well as weight reduction (using respective DiGA). Suggestions of interviewees include further optimization of usability, systematic further training of physicians on DiGA, and the expansion of gamification elements. Physicians have so far lacked well-founded information about DiGA.This indicates that providers likewise need to be empowered to prescribe health apps and DiGA and assess the benefits [11].

International results underline the findings that the use of health apps is significantly less likely among older than younger people [12]. Health apps tend to be used by younger, educated individuals with digital health literacy, while older age and low digital health literacy are associated with non-use of health apps [13]. The authors concluded that the benefits of expanding digital health offerings do not reach vulnerable groups such as the older population that would need to be focused on. Based on current data on the use of DiGA in Germany, it is known that the frequency of DiGA use is significantly lower after the age of 60 [14]. The barriers for older adults with depression to use DiGA should thus be taken into account. Higher age was found to be a decisive predictor of low digital health literacy in a comprehensive study of digital health literacy among the population in Germany [15]. Digital health literacy is the ability to find, understand, assess, evaluate, and apply health-related information in relation to digital health and information services [16]. It combines traditional, health, information, science, media, and computer literacy [17] and can therefore be understood as a combination of health and media competencies. Older persons thus might have a high need for assistance and guidance in (starting) DiGA use. In this context, a secondary analysis of the EVIDENT study, exploring the efficacy of a DiGA in addition to treatment as usual, revealed interesting results. Older age and higher depressive symptoms were associated with higher usage time. Higher adherence predicted a greater reduction in depressive symptoms over 12 weeks. However, these results could mostly be explained by receiving support [18]. In the context of older patients, studies of applicants showed that in the course of installing and administering digital consumer health-related products, friends or family members were consulted in a large number of cases [19]. Prescribing and empowering independent use are therefore associated with challenges for patients and providers [20]. In practice, it is crucial not to leave patients alone after the prescription, but to ensure in close contact that the novel application is used and that this is done in the intended manner [21].

It is noteworthy, the above-mentioned EVIDENT study examined persons up to 65 years of age. Results thus refer to younger age ranges among older adults. Likewise, the focus in the investigation of apps on depression is mainly on younger individuals [22, 23]. The participants in the studies examining the efficacy of the two approved DiGA [24, 25] were up to 65 years of age. Adults aged 65 years and older might need even more support to be able to use a DiGA.

With the intervention of a digital nurse, we want to create a standardized offering in our study for all those adults aged 60 years or older, similar to the above-mentioned frequently used support from relatives. The digital nurse is a person who has been trained to support older people in the commissioning and use of DiGA. Studies investigating the efficacy of support in the use of health apps usually focus on therapeutic support and aspects of motivation for regular use rather than on technical support to enable access to and usability of the prescribed DiGA in the first place. Evidence on the value of digital nurse support aiming at improving the usability of DiGA in older adults is lacking.

### Objectives {7}

Against this background, the primary objective is to evaluate whether it is possible to increase the usage rate of a DiGA with the support of a digital nurse in a population of patients aged 60 or above with depressive symptoms. Further objectives are to improve patient-relevant outcomes, to explore the feasibility of recruitment and intervention in different settings, and finally to explore factors moderating the effect or predicting DiGA use in the target group.

### Trial design {8}

The main hypothesis of the trial is: The support of digital nurses in the process of prescribing a DiGA leads to a higher percentage of DiGA use in older patients with depressive disorders compared to treatment as usual. This hypothesis will be tested in a multi-center randomized superiority trial (RCT) with two equally sized arms and randomization on patient level. This RCT is used for investigation of the feasibility of recruitment and implementation, and proof of concept.

The intervention consists of the additional offer of a digital nurse (intervention group) compared to treatment as usual (control group) in patients who are prescribed a DiGA. The success of the intervention will be measured 8 weeks after randomization. A pilot study preceding this RCT aimed to identify the most appropriate DiGA for the target population that would subsequently be used in the RCT. The pilot study is described in a separate study protocol [9]. DiGA are generally subject to full reimbursement by the German statutory health insurance after prescription. Participation in the trial is cost-free. Access to the DiGA is generally valid for 3 months after the start of DiGA processing. If interested, a re-prescription can also be made by the supervising physician after the end of the study. The support provided by the digital nurse ends at the end of the study period.

## Patient involvement

Patients were involved in the design of the RCT with regard to the choice of the DiGA. Patients tested and evaluated the DiGA available for the target group and thus made a significant contribution to the potential success of the interventions. There was no further public involvement.

## Methods: participants, interventions, and outcomes

### Study setting {9}

Recruitment will take place across sectors (outpatient and inpatient) in three academic hospitals (geriatrics, psychiatry, and psychosomatics) and selected, collaborating general practitioner practices in North Rhine-Westphalia, Germany.

### Eligibility criteria {10}

#### Inclusion criteria

The aim is to recruit a heterogeneous patient population to investigate possible influencing factors and moderators on DiGA use. During the recruitment process, detailed documentation of the screened patients will take place to investigate factors for non-participation.

To be eligible to participate in this study, an individual must meet all the following criteria:

- Provision of signed and dated informed consent form
- Age ≥ 60 years
- Mild to moderate depressive disorder based on ICD-10 codes F32.0, F32.1, F33.0, F33.1
- Access to digital device (desktop computer, tablet, or smartphone) that enables DiGA use
- Stated willingness to comply with all study procedures and availability for the duration of the study

#### Exclusion criteria

An individual who meets any of the following criteria will be excluded from participation in this study:

- Incapacity to consent
- Current suicidality
- Severe auditory and/or visual impairment
- Insufficient German language skills
- Presence of moderate to severe dementia
- Presence of a bipolar disorder, schizophrenia, or other severe psychiatric illnesses that make participation impossible
- Advanced somatic terminal illness
- Current participation in another intervention study
- Current or former use of any DiGA for depression

### Who will take informed consent? {26a}

After pre-screening of eligibility (see item 15, recruitment), final eligibility of patients ≥ 60 years with a mild or moderate depression is assessed during the baseline study visit. If a patient is eligible, a clinician informs the patient in detail about the study and study participation. The patient will be given time to read the patient information and consent material and to ask questions. If the patient is willing to participate, written informed consent is provided.

### Additional consent provisions for collection and use of participant data and biological specimens {26b}

No additional consent provisions for the collection and use of participant data and biological specimens are necessary.

## Interventions

### Explanation for the choice of comparators {6b}

The control intervention is treatment as usual. After DiGA prescription, patients receive a short information by their treating physician, and the manufacturer’s information film about the DiGA is shown. The further process of activating the DiGA and its use are the responsibility of the patient.

### Intervention description {11a}

In addition to a short information by the treating physician after DiGA prescription and the manufacturer’s film, the intervention group receives the offer of regular individual support by a digital nurse who accompanies and supervises the process of request to the health insurance companies, the installation of the app/navigation to the website until the first use of the DiGA up to 8 weeks according to the needs of the patients. The consultations are offered in different ways depending on the patient's needs: by telephone, via video call or face to face. Support is actively offered to patients at least once a week. The digital nurses are trained particularly in the process of prescription and establishing contact with the DiGA manufacturer and the health insurance company. Furthermore, the digital nurses are trained in the installation and application of the DiGA. Within the counseling process, the digital nurses should respond attentively and sensitively to the needs of the participants. They will therefore be trained in motivational interviewing techniques like active listening and change talk. The digital nurses also monitor the emotional state of the participants in order to identify mental crises and suicidal tendencies, that might necessitate intensive care in inpatient settings as well as exclusion from the study.

A guideline is developed that defines the counseling process and the care options. Checklists will be used to ensure fidelity to the guideline. In order to develop best practice guidelines number of sessions, mode and duration of intervention, total support time, and discussed topics will be documented during each counseling session.

The trained digital nurse supports the individual participant in using the DiGA for up to 8 weeks or until the first use of the DiGA, whichever comes first (intervention phase). This means that at the time the participant is able to start the first session of the DiGA, the support of the digital nurse ends. However, the digital nurse will be available to patients until week 16 if they have any problems or questions regarding the DiGA application (follow-up phase).

### Criteria for discontinuing or modifying allocated interventions {11b}

Participants are able to discontinue the study intervention at any time without giving reasons and without suffering any disadvantage, as stated to them prior to informed consent. The study intervention will be discontinued, if it becomes apparent that the system requirements for using the DiGA are not met. Furthermore, study intervention (seeking contact through the digital nurse) will be stopped, if it becomes apparent that the patient's mental or somatic health has deteriorated significantly, and the patient is currently unable to use the DiGA.

When a subject discontinues from the study intervention but not from the study, the remaining study assessments will be completed as indicated by the study protocol.

The data to be collected at the time of study intervention discontinuation will include the following:

- Point in time
- The reason(s) for discontinuing the intervention.
- If possible, the primary endpoint and depressive symptoms at the time of discontinuation.

Thereafter, the participant will be included in all future scheduled assessments, even though not participating in the intervention.

### Strategies to improve adherence to interventions {11c}

During the intervention phase, there are no restrictions on the number, duration, or type of contacts (telephone, videocall, face to face). The meetings are deliberately organized according to the patients’ needs in order to improve adherence. However, the digital nurse will contact the patient at least once a week at the beginning of the intervention period.

The mode of administration, number, and total support time for each participant will be documented and evaluated.

### Relevant concomitant care permitted or prohibited during the trial {11d}

Since DiGA can be used as an add-on therapy to the best medical care, all concomitant therapies are allowed. As the secondary endpoint of depressive symptoms (PHQ-9) might be influenced by concomitant psychotherapies or the use of antidepressants, the number and/or dose of these therapies is recorded.

### Provisions for post-trial care {30}

The possibility to use the prescribed DiGA does not end at the end of the follow-up period. The DiGA may be used by the patients as long as desired. If they wish to use the DiGA for longer than the intended duration of use (prescription valid for 90 days), patients must obtain a follow-up prescription themselves. Participating patients also have the opportunity to contact the clinics and practices after their participation in the study has ended.

### Outcomes {12}

The primary endpoint is DiGA use at week eight after randomization (yes/no). It is recorded whether the patients have started using the DiGA within 8 weeks, i.e., whether they have started at least the first online session. We chose an intervention period of 8 weeks because administrative processes such as request to the health insurance companies, and the installation/navigation to the website may last several weeks until the first use of the DiGA is possible at all.

Secondary endpoints are DiGA use after 16 weeks, change in depressive symptoms (measured by PHQ-9 [26]), and change in treatment of mental disorder after 8 and 16 weeks. The usability of the DiGA will be assessed by the System Usability Scale (SUS) [27] and the User Experience Questionnaire (UEQ) [28] at week eight and 16.

All outcomes will be assessed via personal/telephone interviews and patient self-report.

### Participant timeline {13}

The participant timeline is shown in the table below [26–31].

**Table:**
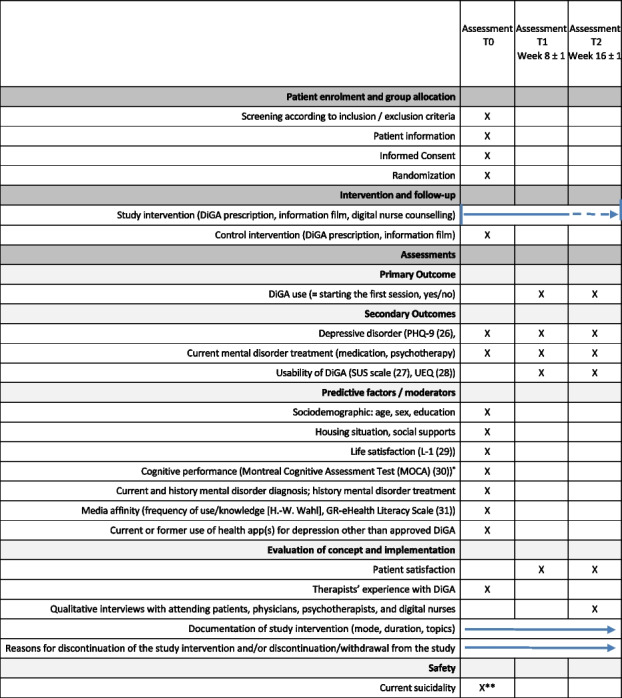


*If organizationally possible, **is only asked again if the baseline assessment is scheduled on a different day than the patient's inclusion to the study. Otherwise, suicidality is already queried during the enrolment process

### Sample size {14}

The sample size to test the main hypothesis was calculated using a Fisher’s exact test at a one-sided significance level of 5% and to achieve a power of 80%. It was assumed that under control 30% of the participants would start using DiGA and that this proportion would be increased to 50% through the support of the digital nurse. This resulted in a required number of cases of 84 patients per group. Due to a possible drop-out (assumption approx. 15%), 100 patients per group should be included in the study.

### Recruitment {15}

Recruitment will take place at all three participating hospitals (see 9.), as well as at selected primary care practices. For recruitment in the participating hospitals, patients can be recruited from the outpatient clinic, the ward, and the day clinic. Due to different populations and settings, recruitment procedures will differ between hospitals and primary care practices, inpatient and outpatient settings in hospitals, and even between the participating hospitals themselves. Recruitment should fit as well as possible into the respective clinic’s and practice’s daily routine to ensure feasibility and success. The procedure described below will therefore be adapted to the conditions in the respective setting, and screening manuals will be written.

#### General recruitment procedure

##### Step 1: eligibility screening

Patients will be recruited as they visit the clinic (inpatient or outpatient) or practice. First, patients are screened regarding age. Those aged ≥ 60 years are further screened with regard to mild or moderate depression based on ICD-10 codes F32.0, F32.1, F33.0, and F33.1. No restrictions about gender, race and ethnicity, or upper age limit are made. However, in order to use all survey instruments, a restriction to sufficient German language skills is necessary. Thus, sufficient knowledge of the German language and the ability to give informed consent is judged. These patients are invited to participate in the study and the final eligibility screening.

##### Step 2: final eligibility screening

Depending on the recruitment setting eligible patients are approached by the general practitioner (GP)/clinician or study nurse for final eligibility assessment. If a patient is eligible according to all criteria mentioned above, the patient is invited to participate in the study and receives the DiGA prescription. After the provision of written informed consent (see item 26a above), participants are then randomized to the intervention and control group. If necessary, a new appointment to start the intervention is made.

##### Step 3: start of intervention

The intervention starts with a short information film. Participants of the intervention group additionally have their first appointment with the digital nurse afterwards.

## Assignment of interventions: allocation

### Sequence generation {16a}

To guarantee adequate sequence generation and allocation concealment, centralized online randomization with 1:1 allocation to intervention or control group is done through REDCap. Permuted blocks of random length are used and are stratified by the center to guarantee nearly equal group size within each center.

### Concealment mechanism {16b}

Allocation concealment will be ensured, as the software will not release the randomization code until the patient has been entered into the trial, which takes place after all baseline measurements have been completed.

### Implementation {16c}

A person at the Department of Medical Informatics, Biometry, and Epidemiology (AMIB) not further involved in the study creates the allocation sequence which will be implemented in REDCap; clinicians and study nurses will enroll participants and will assign them to the interventions accordingly.

## Assignment of interventions: blinding

### Who will be blinded {17a}

It is not feasible nor possible for the participating patients and most project staff to be blinded to treatment allocation. Primary outcome assessment will be based on patient report, blinding is not possible. Staff who analyze the data will be blinded (arms will be designated randomly at that point as A and B). Data review will be performed blinded.

### Procedure for unblinding if needed {17b}

The trial design is open-label, and therefore, there is no unblinding procedure.

## Data collection and management

### Plans for assessment and collection of outcomes {18a}

Data collection will be the responsibility of the clinical trial staff under the supervision of the site investigator. The investigator will be responsible for ensuring the accuracy, completeness, legibility, and timeliness of the data reported.

In accordance with the clinical routine, study visits will take place on-site at the clinic or practice, study data will be captured on printed Case Record Forms (CRF), and then entered in the corresponding electronic (e)CRF as soon as possible or captured directly through the eCRF (see the “Data Management {19}” section). Patient questionnaires will be filled out during the waiting times of the visits. Most questionnaires used are validated (see the “Outcomes {12}” section). Prior to the start of the study, the leading site investigator will instruct all study personnel on study procedures and the study-related data collection process.

The diagnosis of depression, possible bipolar disorder, schizophrenia, moderate or severe dementia, or other severe psychiatric disorders (exclusion criteria) is made by the treating physician or psychotherapist. The assessment of cognitive performance (MOCA) is made by the treating physician or psychotherapist and/or trained study nurse as far as this is possible in the course of medical care. It is to be expected that MOCA will be collected in inpatient clinics in particular. The assessment of German language skills and possible auditory and/or visual impairments will be made by the recruiting physician at his/her own discretion. The patient is asked about comorbidities, including terminal illness, current or former use of a prescribed depression DiGA, and participation in other studies.

After inclusion in the study, baseline data and baseline questionnaires are collected. Patient-reported data is collected via an online survey using a tablet or by face to face interviews by the study personnel. The assessments after 8 and 16 weeks are not planned at present as all outcomes are purely self-reported by the patients. These data will be collected via telephone interviews with the study personnel or via an online survey.

The information of the support provided by the nurse will also be collected as part of the study and will form the basis for the development of a standardized support concept by the digital nurse, which will then be investigated in multi-center studies.

### Plans to promote participant retention and complete follow-up {18b}

At study inclusion, patients can choose their mode of follow-up assessments. They can choose between contact by the study personnel to assess data via interview or getting a link to the eCRF on their mailing address. If a participant chooses the second option but does not complete the eCRF within the timeframe provided, the center will contact the participant by telephone and offer help and counseling to finish the assessment.

If a patient misses one scheduled assessment, the following actions must then be taken:

- The site will attempt to contact the participant, within 1 week, counsel the participant on the importance of maintaining the assigned assessments and, if possible, will perform the assessment by telephone. Furthermore, the site will ascertain if the participant wishes to continue in the study.
- Before a participant is deemed lost to follow-up, the investigator or designee will make every effort to regain contact with the participant (where possible, five telephone calls, or three calls, and two certified letters to the participant’s last known mailing address). These contact attempts will be documented in the participant’s medical record or study file.
- Should the participant continue to be unreachable, he or she will be considered to have withdrawn from the study with a primary reason of lost to follow-up.

### Data management {19}

The clinical trial staff at the participating centers under the supervision of the site investigator will be responsible for data entry. The investigator will be responsible for ensuring the accuracy, completeness, and timeliness of the data reported. All source documents will be completed in a neat, legible manner to ensure accurate interpretation of data. Clinical data will be entered into REDCap (Research Electronic Data Capture), a system provided by the AMIB [32, 33]. REDCap is a secure, web-based software platform designed to support data capture for research studies. It provides 1) an intuitive interface for validated data capture; 2) audit trails for tracking data changes and exports; 3) automated export procedures for seamless data downloads into common statistical packages; and 4) procedures for data integration and interoperability with external sources. The data system provides internal quality checks, such as automatic range checks, to identify data that appear inconsistent, incomplete, or inaccurate. Prior to the start of recruitment, the staff in the clinics and practices involved in this study receives training regarding data entry and management using REDCap.

Data capture and processing will be in accordance with the applicable law on personal data protection and with the GDPR (EC) 2016/679 of the European Parliament and of the council. Access to the data is strictly limited to authorized persons. Data are protected against unauthorized access. The database is hosted by the AMIB on servers, which are located at the RUB in access-protected server rooms.

Personal data collected for this study will be stored for 10 years from the completion of the study. When the study is completed, access to study data will be provided upon request to the PI.

### Confidentiality {27}

All data will be initially collected by investigators in the recruiting trial sites. Together with information on the trial, eligible patients will be informed about data capture, transmission, analysis processes, and their rights according to the General Data Protection Regulation (GDPR). Once a patient is eligible and has given his/her informed consent (concise version) to trial participation and data collection, the investigator will assign with the help of a separate “contact database” the patient a unique patient identification code. Patient identification code lists will be filled at the recruiting sites, and are part of the investigator site file. These lists are the only documents that allow for re-identification of the patients and remain at the recruiting site. Access is only possible for authorized persons of the study team. All clinical data entered by the investigators (or their designated staff) into eCRFs will be recorded in an anonymized form exclusively using the patient’s identification code.

Patients may withdraw their informed consent. If patients withdraw their consent, no further data will be collected. However, the data processing carried out up to the date of withdrawal remains lawful. If the informed consent is withdrawn, the patient has the right of data deletion according to the GDPR. Information as to when and why a patient was randomized and when he withdrew consent must be retained in the documentation. According to GDPR, the institution responsible for conducting the study is generally obliged to delete its data from the study database after the withdrawal of consent.

### Plans for collection, laboratory evaluation, and storage of biological specimens for genetic or molecular analysis in this trial/future use {33}

Not applicable.

## Statistical methods

### Statistical methods for primary and secondary outcomes {20a}

We assume that the additional support from a digital nurse will lead to a greater proportion of older patients using the DiGA compared to support from an explain film alone.

Since this is a feasibility and proof-of-concept study, the main hypothesis will be tested one-sided at a significance level of 5%, i.e., the null hypothesis “smaller or equal frequency of use in the intervention group compared to the control group” vs. the alternative hypothesis “greater frequency of use in the intervention group compared to the control group” will be tested, i.e., the following null and alternative hypothesis will be tested:

$$H_0:p_I\leq p_{K\;}vs.\;H_1:p_I>p_K.$$

Here, ${p}_{I}$ and ${p}_{K}$ stand for the frequency of DiGA use in the intervention (digital nurse) and control group, respectively.

#### General approach

Statistical analysis will be performed using the statistical software R (www.r-project.org), version 4.1.0 or higher. Data scientists will remain blinded through the use of non-meaningful group labels, e.g., the groups will be called “group A” and “group B”. For descriptive statistics, continuous variables will be summarized using the arithmetic mean and standard deviation; in case of skewed distribution, median and interquartile range are used. Categorical variables will be summarized using total counts and percentages.

Unless otherwise stated, the estimate with the corresponding two-sided 95% confidence interval and two-sided *p*-value will be reported for all confirmatory and exploratory analyses. *P*-values less than 5% will be considered statistically significant. Details of the statistical analysis will be later specified in the statistical analysis plan (SAP).

Demographic and baseline variables will be summarized in a table, stratified by treatment and control group. Continuous variables will be summarized using the arithmetic mean and standard deviation; in case of skewed distribution, median and interquartile range are used. Categorical variables will be summarized using total counts and percentages. No inferential statistics will be used to compare baseline characteristics. Details on variables reported in this table will be defined in the SAP.

#### Primary and secondary endpoints

For the primary endpoint, the main aim is to estimate the odds ratio (OR) of DiGA use with digital nurse support vs. unsupported prescriptions. The confirmatory analysis will be conducted in the full analysis set (FAS, see below) based on the intention-to-treat (ITT) principle. The analysis of the primary endpoint is carried out using a logistic regression model. In addition to the intervention group, the model also includes age, gender, and the recruiting center as well as possible other factors as covariates. One-sided *p*-value corresponding to the hypothesis given above, estimated OR and corresponding one-sided confidence interval will be reported. One-sided *p*-value will be calculated as half of the *p*-value from the fitted model output.

Analyses of all secondary outcomes are exploratory and will be done as complete case analyses, meaning that only cases with available data for the analysis are included. Analysis of the secondary efficacy outcomes will be undertaken following the same framework as the primary outcome model, particularly using the same covariates in the models. For each continuous outcome including the PHQ-9 and SUS, a linear regression model will be fitted. For the analysis of PHQ-9, the baseline value is included in the model as an additional covariate. The estimates for the group effect with accompanying 95% confidence intervals and two-sided *p*-values will be reported. Details of the models will be specified in the statistical analysis plan (SAP).

### Interim analyses {21b}

This trial is not planned as a group-sequential or adaptive trial. Hence, we have no interim analyses.

### Methods for additional analyses (e.g., subgroup analyses) {20b}

Instead of subgroup analyses, explorative moderator analyses are planned. Possible moderators for DiGA use are sex, age, severity of depressive symptoms, treatment setting, education, media affinity, eHealth literacy, cognitive function, and current or former use of health apps or DiGA. Moderator analyses will be performed using the logistic regression model from the primary endpoint analysis with an additional inclusion of the treatment x moderator interaction.

All moderator analyses are exploratory. Further exploratory analyses are done to identify predictors of DiGA use. This analysis will be done only in the control group.

Feasibility of a multi-center trial will be explored by consideration of recruitment rates, observed difference in depressive symptoms (PHQ-9), and patient’s satisfaction with the intervention.

### Methods in analysis to handle protocol non-adherence and any statistical methods to handle missing data {20c}

Two differing data sets are defined for the analyses:

#### Full analysis set (FAS)

The FAS, based on the ITT strategy, is defined as all randomized patients according to their allocated intervention group, who fulfill the inclusion criteria and do not fulfill any exclusion criteria.

#### Safety analysis dataset

The safety analysis set will include all randomized patients.

Missing values of the primary endpoint will be imputed. Details of the model, imputation of missing values and sensitivity analysis will be specified in the SAP.

### Plans to give access to the full protocol, participant-level data, and statistical code {31c}

Full study protocol will be published in an international scientific journal. Study results will be published, regardless of whether the results are positive or negative, according to the Consolidated Standards of Reporting Trials (CONSORT). The SAP will be published as an appendix in the publication of results. Access to the participant-level dataset and statistical code will be given upon request after analyses have been finished.

## Oversight and monitoring

### Composition of the coordinating center and trial steering committee {5d}

The overall coordinating center is the Institute of General Practice and Family Medicine (AM RUB) of the Ruhr-University Bochum; the head of the department is the head of the DiGA4Aged consortium. A trial steering committee will be established to provide expert advice and oversight and ensure that the trial is conducted to the required standards. The steering committee includes the respective leading investigator for the setting of General Medicine, Geriatrics, Psychosomatics (PI of the RCT), and Psychiatry as well as Biometry. The steering committee is responsible for agreement of the final protocol, protocol changes, and reviewing progress throughout the study. Scientific assistants and digital nurses control the trial progress on a day-to-day basis.

### Composition of the data monitoring committee, its role and reporting structure {21a}

Since this study is being conducted with an approved DiGA, it is a low-risk study, so only reduced monitoring will be done. Each clinical site will perform internal quality management of study conduct, data collection, documentation, and completion. Quality control procedures will include:

Informed consent—Study staff will review both the documentation of the consenting process as well as a percentage of the completed consent documents. This review will evaluate compliance with good clinical practice, accuracy, and completeness. Feedback will be provided to the study team to ensure proper consenting procedures are followed. A data monitoring committee is not needed, see item 22 below.

### Adverse event reporting and harms {22}

This trial investigates the possible increase in DiGA use due to a digital nurse. The DiGA is listed in the DiGA register and can be prescribed without any safety concerns. The intervention itself therefore is relatively low risk. This is a trial to investigate feasibility and proof of concept. We do not expect significant adverse effects arising from the trial itself. We have therefore decided not to have a separate data monitoring and safety board. Oversight of the trial will be managed by the trial steering committee.

### Frequency and plans for auditing trial conduct {23}

At the beginning of the study, the whole consortium including the trial steering committee (see {5d} above) will meet every 2 weeks in order to properly initiate the trial and review trial conduct. During the recruitment phase, the meetings then take place monthly. The project management and digital nurses meet every 2 weeks in order to discuss trial progress and problems. The recruitment process is reported every month. Recruitment figures are continuously compared with the target; if necessary, measures to increase patient numbers can be discussed in the management team on an ongoing basis. Plausibility checks of the data are already carried out in the course of the study and corresponding queries are sent to the centers to ensure high data quality. Further aspects of internal data monitoring and quality control have been described in section {21a}. External audits are not planned.

### Plans for communicating important protocol amendments to relevant parties (e.g., trial participants, ethical committees) {25}

The project management communicates the necessary amendments to the study protocol with the responsible ethics committees and, if approved, updates the information in the clinical trials registry. The amendments are communicated to the leading investigators of the four participating study centers including the PI. The site investigators are responsible for communicating the amendments to the study personnel involved and, where relevant, also to participants of the respective study site.

### Dissemination plans {31a}

The results will be published in a final report in accordance with the sponsor’s guidelines. In addition, scientific results will be disseminated through publications in peer-reviewed scientific journals that comply with the International Committee of Medical Journal Editors’ guidelines for authorship and through presentations at national and international scientific conferences.

## Discussion

The use of digital health interventions in Germany is growing but has room for improvement. Older adults use health apps and DiGA less frequently than younger adults, probably due to reduced health literacy and digital skills, and lack of support. Previous studies suggest that mobile apps can be helpful in reducing care and symptom severity in somatic and mental disorders [3]. However, it has also been shown that adherence can be significantly improved through support [34]. With the rise of digitalization in the healthcare system, the implementation of a trained digital nurse is an exciting path that ideally also saves time for the treating physicians.

The results of this RCT study will give insights into the feasibility of recruiting adults with depression aged 60 years and older for the use of a depression DiGA. As a proof of concept study, this trial will also give evidence of the efficacy of support by a digital nurse in using the prescribed DiGA. Factors that might moderate the effect, such as sociodemographic factors, living situation and assistance, media affinity, or digital health literacy will be explored.

Qualitative and quantitative evaluations of the trial implementation will result in best practice guidelines on how to support and improve DiGA prescription and successful use by older adults with mild to moderate depression. Barriers and facilitators of digital nurse support in older adults with mental illness will be revealed and compared to existing literature [34]. Moreover, the results of this study will be used to elaborate an overarching concept for successful digital nurse support for older adults receiving a DiGA prescription, regardless of the underlying disease.

This study aims to investigate whether the use of digital nurses to support patients in installing and starting DiGA treatment is successful. If so, this would strongly support the implementation of digital nursing in clinical routine. These could be medical assistants or digital-savvy students who receive a short training course. This could optimize medical resource consumption and at the same time strengthen the therapeutic benefits of DiGA. This concept will include a discussion on the implementation against the background of the current health care in Germany and underlying statutory regulations, possibilities of financing a digital nursing concept in vulnerable populations, and the required qualification of non-medical staff in order to deliver digital support.

## Trial status

Recruiting started in July 2024. The current protocol is version 4.0 of 15–07–2024. Patient recruitment is estimated to be completed around July 2025.

## Data Availability

Data collected for this study will be analyzed and stored at the AMIB for 10 years from the completion of the study. When the study is completed, access to study data will be provided upon request to the principal investigators.

## Abbreviations

AMIB: Department of Medical Informatics, Biometry and Epidemiology
CONSORT: Consolidated Standards of Reporting Trials
CRF: Case Report Form
DVG: Digitales Versorgungsgesetz (Digital Health Care Act)
DiGA: Digitale Gesundheitsanwendung (digital health application)
eCRF: Electronic Case Report Forms
FAS: Full analysis set
GCP: Good Clinical Practice
ICH: International Council on Harmonisation
ITT: Intention-to-treat
MOP: Manual of Procedures
PHQ-9: Patient Health Questionnaire-9
PI: Principal Investigator
UEQ: User Experience Questionnaire
SAP: Statistical analysis plan
SUS: System Usability Scale
SOP: Standard Operating Procedure
